# Influence network model uncovers relations between biological processes and mutational signatures

**DOI:** 10.1186/s13073-023-01162-x

**Published:** 2023-03-06

**Authors:** Bayarbaatar Amgalan, Damian Wojtowicz, Yoo-Ah Kim, Teresa M. Przytycka

**Affiliations:** 1 National Center for Biotechnology Information, National Library of Medicine, National Institutes of Health, 8600 Rockville Pike, 20894 Bethesda, USA; 2 Current address: Faculty of Mathematics, Informatics, and Mechanics, University of Warsaw, ul. Banacha 2, 02-097 Warszawa, Poland

**Keywords:** Mutational signatures, Networks, Causality inference, Mutational processes in cancer, Breast cancer, APOBEC

## Abstract

**Background:**

There has been a growing appreciation recently that mutagenic processes can be studied through the lenses of mutational signatures, which represent characteristic mutation patterns attributed to individual mutagens. However, the causal links between mutagens and observed mutation patterns as well as other types of interactions between mutagenic processes and molecular pathways are not fully understood, limiting the utility of mutational signatures.

**Methods:**

To gain insights into these relationships, we developed a network-based method, named GeneSigNet that constructs an influence network among genes and mutational signatures. The approach leverages sparse partial correlation among other statistical techniques to uncover dominant influence relations between the activities of network nodes.

**Results:**

Applying GeneSigNet to cancer data sets, we uncovered important relations between mutational signatures and several cellular processes that can shed light on cancer-related processes. Our results are consistent with previous findings, such as the impact of homologous recombination deficiency on clustered APOBEC mutations in breast cancer. The network identified by GeneSigNet also suggest an interaction between APOBEC hypermutation and activation of regulatory T Cells (Tregs), as well as a relation between APOBEC mutations and changes in DNA conformation. GeneSigNet also exposed a possible link between the SBS8 signature of unknown etiology and the Nucleotide Excision Repair (NER) pathway.

**Conclusions:**

GeneSigNet provides a new and powerful method to reveal the relation between mutational signatures and gene expression. The GeneSigNet method was implemented in python, and installable package, source codes and the data sets used for and generated during this study are available at the Github site https://github.com/ncbi/GeneSigNet.

**Supplementary Information:**

The online version contains supplementary material available at 10.1186/s13073-023-01162-x.

## Background

Traditionally, research in cancer genomics has been focused on the identification of cancer driving mutations that confer a growth advantage to cancer cells. However, since cancer often emerges as a byproduct of various mutagenic processes such as UV light or a faulty DNA repair mechanism, cancer genomes also accumulate numerous mutations with seemingly no direct roles in carcinogenesis. Different mutagenic processes often lead to distinct patterns of somatic mutations called *mutational signatures*. This provides an opportunity to leverage such signatures for studying interactions between mutagenic processes and other cellular process.

Starting from the pioneering work of Alexandrov et al. [1], several computational methods have been developed to infer mutational signatures. The exposure of a genome to a given mutagenic process is measured by the number of mutations attributed to the corresponding signature. While the relations between some of these computationally-derived signatures and mutational processes causing them could be established based on prior knowledge or association with specific environmental or molecular factors [2, 3], the etiology of many signatures remains unknown or not fully understood. Mutagenic processes can be caused by perturbations of molecular pathways, and vice verse, they can disrupt normal cell function. Therefore elucidating the relation between mutational processes and activities of molecular pathways is of fundamental importance for a better understanding of the etiology of mutagenic processes and their role in carcinogenesis. Gene expression provides the most accessible measurement of the activities of cellular processes. Therefore, we reasoned that interrogating the relation between gene expression and exposure of a mutational signature might provide important clues on both: the etiology of mutational signatures and on the impact of the corresponding mutational processes on cellular functions.

There are several know examples where deficiencies in the activities of some genes such as MUTYH [4], ERCC2 [5], MSH6 [6], and FHIT [7] have been linked to specific signatures. Extending this observation beyond individual genes, a recent study successfully linked exposures of mutational signatures to mutated subnetworks [8]. In addition, a correlation between the expression of the APOBEC family of genes and the exposures of signatures SBS2 and SBS13 (so-called APOBEC mutational signatures) has frequently been observed [2, 9]. Finally, a perturbation of some cellular processes such as DNA replication or repair are known to be mutagenic.

There are also many examples of the reverse relation. For instance, tobacco smoking is not only mutagenic but it is also believed to activate the immune response [10]. Specific mutagenic processes are known to activate specific DNA repair pathways. In addition, a mutagenic process can cause a cancer-driving mutation [11, 12, 13 which can, in turn, cause changes in gene expression.

Finally, mutagenic processes themselves have been known to interact with each other. For example, homologous recombination deficiency (HRD) is often accompanied by mutational signatures related to APOBEC activity [8, 14].

Applying a clustering-based method to genome-wide expression and mutational signature data, Kim et al. identified coherently expressed groups of genes associated with specific combinations of mutational signatures, providing interesting insights regarding these signatures [8]. However, while such cluster-based analysis can suggest associations between signature exposures and the activities of biological processes, it lacks the ability to provide more precise explanations.

**Fig. 1:**
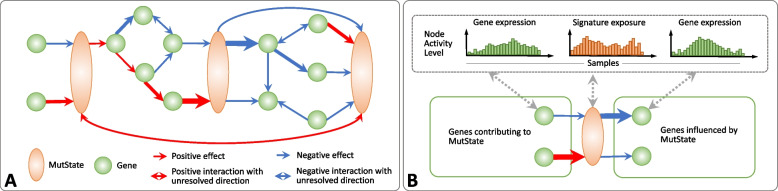
Gene-Signature Network (GSN). **A** GSN is a weighted-directed network consisting of two types of nodes: genes (green circles), and MutStates (orange ovals) corresponding to signatures. Edges of a GSN represent inferred influences and might be either positive (red) or negative (blue). **B** Edges of a GSN are inferred based on the activities of nodes (gene expression for genes and signature exposures for MutStates (top panel)). A directed edge from a gene to a MutState represents a putative influence from the gene to the MutState. Analogously, a directed edge from a MutState to a gene represents an inferred effect of the MutState on the gene. The thickness of the arrows reflects the edge weights

To fill this gap, we introduce a network-based method, named GeneSigNet (*Gene* and *Sig*nature Influence *Net*work Model), aiming to uncover interactions among mutagenic and cellular processes, and to provide insights into mutagenic processes underlying individual signatures. Utilizing gene expression and mutational signature data from cancer patients, GeneSigNet constructs a Gene-Signature Network (GSN) that represents asymmetric dependencies among two types of node entities — genes and MutStates. MutState nodes are in one-to-one correspondence with mutational signatures and each MutState represents an abstract (directly unobserved) cell state associated with the corresponding mutational signature (for a detailed description, see the “Results” section: The GeneSigNet method). Both types of nodes have patient-specific activities: gene expression for nodes corresponding to genes and signature exposure for nodes corresponding to MutStates. GeneSigNet utilizes these activities to infer directed edges between nodes in the GSN (Fig. 1). The interactions represented by these edges correspond to potentially indirect dependencies between activities of network nodes. Ideally, we would like to infer causal relationships between molecular activities and mutational signatures. However, in complex molecular systems, inferring causality is one of the most challenging open problems. In some settings, prior information about gene function (such as transcription factor versus potential target gene) or special type of data such as response to a perturbation of the expression of a gene can be leveraged to infer causality [15, 16, 17, 18]. In our setting, however, no perturbation data or prior knowledge is available, requiring GeneSigNet to rely solely on the observed activities of nodes and infer network edges representing the relations between the nodes and their dependency directions. While inferring directed influences without such additional information remains challenging, significant efforts have been made to identify the conditions where the direction of an influence can be inferred with a reasonable level of success. For example, independent component analysis-based method LiNGAM allows to discover the causal relations between non-Gaussian random variables when the number of variables is much smaller than the number of observations [19]. A node-ordering heuristic, GeneNet [20] constructs a directed acyclic causal network by first computing a partial correlation network and subsequently selecting directed edges from the network based on a multiple testing of standardized partial variances.

Sparse estimation of partial correlations (SPCS) also builds on partial correlation relations, which computes a sparse asymmetric weight matrix representing directed influences among nodes in the network. The sparsity constraints ensure that only a small fraction of influential edges have nonzero weights while many entries of such inferred matrix are set to zero and one-directional [21].

In contrast, the statistical higher moments were used as indicators of the direction of dependency between two variables [22] under the assumption of absence of confounding effects.

The GeneSigNet method builds on the last two approaches. First, a sparse partial correlation technique (SPCS) is used to obtain an initial sparse weighted directed network. We opted to infer a sparse network in order to focus on strongest trends that are more likely to suggest mechanistic explanations. This initial network contains bidirectional edges. Next, where applicable, a novel partial higher moment strategy is used to resolve (orient) bidirectional edges. We note that GeneSigNet does not attempt to construct a fully resolved oriented graph, leaving many edges as bidirectional. We found that this approach compares favorably to the previously proposed techniques that can be used for this task.

We applied GeneSigNet to two cancer datasets, breast cancer and lung adenocarcinoma, for which sufficient numbers of patient samples with gene expression data are available and the interactions of mutational signatures are partially known. For the network reconstruction, we utilized genes in two GO categories: metabolism and immunity. It is increasingly appreciated that cellular metabolic regulation can lead to DNA damage and can impact DNA repair, stimulating interest in the understanding of the cross-talk between DNA-damage and metabolism [23]. In addition, DNA damage response is known to interact with immune system and a better understanding of this interaction can guide cancer immunotherapy [24]. Therefore to empower the method to gain more insights into these important interactions, we limited the set of nodes corresponding to genes to these two categories.

The relations inferred by the GeneSigNet model are overall consistent with current knowledge, but also include several interesting novel findings. In particular, the model suggests a causative relation from the homologous recombination deficiency signature (SBS3) to a clustered APOBEC mutation signature, and also linked Signature 8 (SBS8) to nucleotide excision repair (NER) pathway. The latter connection is consistent with the recent findings based on an experimental study in mouse [25]. In addition, GeneSigNet identified a novel relation between APOBEC hypermutation and the activation of regulatory T cells which presents an important implication in immunotherapy, and captured a relation of APOBEC signature (SBS2) with DNA conformation changes among other findings. Taken together, our results demonstrate that GeneSigNet provides novel and important insights.

## Methods

### The GeneSigNet method

#### Gene-Signature Network

The main idea behind GeneSigNet is a construction of a Gene-Signature Network (GSN) consisting of two types of nodes: nodes corresponding to genes, and nodes corresponding to mutational signatures (Fig. 1A). Patient-specific activity of a node corresponding to a gene is measured by gene expression. The exposure of a mutational signature can be seen as a measure of the activity of the corresponding mutagenic process, such as an exogenous mutagen, or the activity of a cellular process triggered in the response to a mutagen. This motivates the concept of *MutState* defined as an abstract representation of the process(es) effectuating a mutational signature. The activity of this state is measured as the exposure of the corresponding mutational signature in the given patient. Statistical relations on the activities of genes and MutStates are likely to shed light on the genes and pathways associated with, and potentially contributing to, the level of the exposure of the corresponding mutational signature. To this end, GeneSigNet infers directed edges between both types of nodes, utilizing patient-specific gene expression (for genes) and exposures of mutational signatures (for MutStates) as described below (Fig. 1B). Other than the difference in the definitions of node activities (and the subsequent interpretation), the network inference algorithm does not distinguish between the two types of nodes.

#### A high level description of the GeneSigNet inference method

**Fig. 2:**
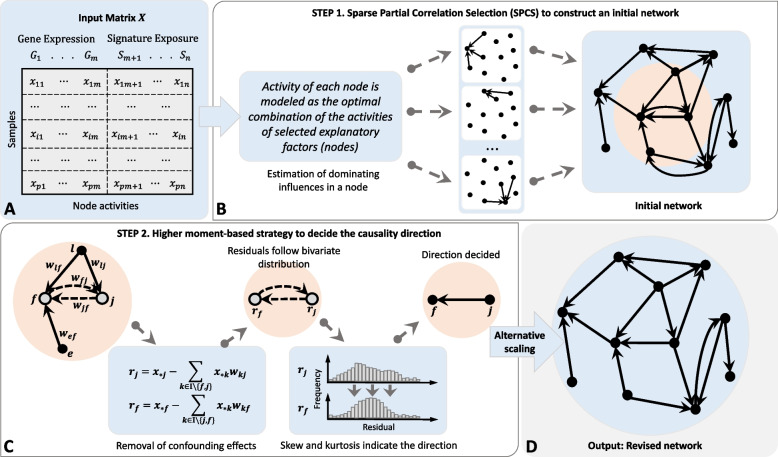
Workflow of GeneSigNet. **A** Input matrix *X* of node activities constructed by concatenating gene expression values (for genes) and signature exposures (for MutStates) across *p* samples (patients). **B** Given the input matrix *X*, we infer a network of *n* nodes. For each node, Sparse Partial Correlation Selection (SPCS) is used to simultaneously estimate the weights of incoming effects from the other $n-1$ nodes. **C** For each bidirectional edge (*j*, *f*), the residual vectors $r_j$ and $r_f$ corresponding to nodes *j* and *f* are obtained by removing effects of the $n-2$ nodes other than the two nodes of the considered edge. The non-zero weight $w_{kf}$ obtained by SPCS denotes the strength of the confounding effect on node *f* coming from node *k*. The direction of a influence effect between the pair of nodes is determined based on the partial higher moment statistics, skewness and kurtosis of residuals $r_j$ and $r_f$. If both moments support the same direction with the heavier partial correlation weight (see Additional file 1: Equation S5), then the edge corresponding to the opposite direction is removed, otherwise, both edges remain in the network. **D** Edge weights are normalized using an alternative scaling algorithm, and the final weighted-directed network is obtained as the output

GeneSigNet method constructs GSN in two main steps: (i) constructing a preliminary directed network using a sparse partial correlation selection (SPCS), and (ii) revising the initial network by testing whether the orientation of bidirectional edges can be decided using a partial higher moment strategy adapted to a network setting. The workflow of the GeneSigNet method is shown in Fig. 2.

In the first step, given the input matrix describing the activities of genes and MutStates across samples, GeneSigNet constructs an initial weighted-directed graph using a sparse partial correlation selection (SPCS) (Fig. 2B). Specifically, considering each node as a target, GeneSigNet uses SPCS to compute the weights of incoming effects from the other $n-1$ nodes. This SPCS step builds on the fact that partial correlations can be approximated by sparse regression coefficients and utilizes a constraint on the $l_1$ norm enforcing the weights of the incoming edges to provide a sparse solution while avoiding over-fitting (see Additional file 1: Sections S1.1 and S1.2, for a detailed description). Sparse correlation techniques lead to a construction of a “sparse” network focusing on the strongest trends that are easier to interpret and are more likely to suggest mechanistic explanations although this is done at the cost of a potential loss of some information (for more discussions, see the “Discussion” section). We note that since SPCS is applied to each node separately, GeneSigNet ensures a locally-sparse solution, rather than constraining the network edges globally.

In the second step, GeneSigNet refines the initial network by reducing the number of bidirectional edges remaining after the first step. The idea is an adaptation of the basic bivariate higher moment strategy to multivariate analysis. Specifically, for any two nodes having potential effects on each other in the initial network (endpoints of a bidirectional edge), we first utilize the partial correlation technique to remove confounding effects due to the presence of the other $n-2$ variables from the observed activities of the two nodes and obtain the residuals representing the remaining dependencies between the pair [26]. Under the assumption that all confounding effects due to the presence of the other $n-2$ variables were successfully removed by partial correlation, the influence variable may be distinguished from the affected variable by comparing the higher moments of the two residual distributions. Specifically, the affected variable is expected to be closer to normality than the influence factor, and the skewness and kurtosis are the higher moment statistics used to measure the close-normality of distributions. We refer to this strategy as *partial higher moment strategy*.

We note that the method relies on simplifying assumptions which might not be fully satisfied in real biologic relations. In particular, the assumption that all confounding effects come from the activities of the remaining $n-2$ nodes in the network is an oversimplification since in such complex systems, potential effects from unobserved latent factors are likely to be present. Thus, we next evaluated the performance of the method on simulated and real data (to the extent possible in the absence of the ground truth).

**Fig. 3:**
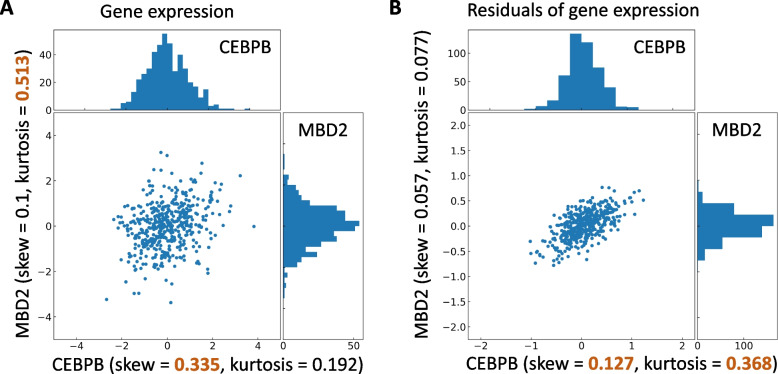
Basic higher moment statistics (**A**) versus the partial higher moment strategy (**B**). The experimentally confirmed regulatory relation is from CEBPB to MBD2. Using uncorrected moment statistics, the two higher moment statistics, skewness and kurtosis, support contradictory directions (**A**). The proposed partial higher-moment strategy predicts the correct direction (**B**). For each case, brown color indicates the higher value for a given moment

Figure 3 provides a real example illustrating the necessity of removing confounding effects before applying the higher moment statistics to correctly indicate the influence direction. Before removing confounding effects, the two higher moment statistics supported contradictory directions (Fig. 3A). After removing the confounding effects, both statistics correctly indicate the influence direction [27] (Fig. 3B). More examples based on experimentally confirmed regulatory directions [27] are provided in Additional file 1: Fig. S2.

Finally, alternate scaling [28], a matrix normalization algorithm, is used to bring the total incoming and outgoing effects of each node into the same range (for details, see Additional file 1: Section S1.4). A detailed description of GeneSigNet is provided in Additional file 1: Section S1.

### Materials

#### Breast cancer data

The normalized gene expression data for 266 breast cancer (BRCA) patients were downloaded from Table S7 in [29]. Gene expression profiles for 2,204 genes involved in either DNA metabolic or immune response processes of the Gene Ontology (GO) database were selected for the analysis.

For mutational signatures, somatic mutation data were downloaded from the ICGC data portal [30]. The 3,479,652 point mutations were assigned to mutational signatures using SigMa [9]. SigMa divided all mutations into two groups, close-by *C*lustered and *D*ispersed mutations, and assigned each of these mutations to one of 12 COSMIC v2 signatures [31] which were previously identified as active in BRCA (Signatures 1, 2, 3, 5, 6, 8, 13, 17, 18, 20, 26, and 30). From the signatures classified by SigMa as described above, signature phenotype profiles 1D, 2C/D, 3C/D, 5D, 8C/D, and 13C/D that had exposure levels of at least 10% within each group were selected for further analysis (the numbering refers to the COSMIC signature index and C/D denotes signatures attributed to clustered and dispersed mutations). Examining their correlation patterns among patients, some of the signatures were grouped as follows: Signatures 3C/D and 8D were combined into DSB (double-stranded DNA break repair) related signatures, and Signatures 2C and 13C/D into APOBEC related signatures. The remaining signatures are treated separately, resulting in Signature 1, 2D, 5, APOBEC, DSB. A log transformation was consequently performed on exposures of each signature to make its distribution shape closer to a bell curve of normality.

Furthermore, we included binary information of homologous recombination deficiency as an additional variable in the analysis. The binary alteration information was obtained by aggregating functional inactivation information for BRCA1/BRCA2 and 16 other HR genes as provided in Supplementary Tables 4a and 4b of Davies et al. [32]. The positive entries were assigned a real value of 4.218 in the SPCS model with the hyperparameter search for the best performance in terms of the means of minimum least square errors and maximum Pearson correlation between responses and predictions over all nodes.

#### Lung adenocarcinoma data

The expression data (RNA-seq) of the lung adenocarcinoma (LUAD) from The Cancer Genome Atlas (TCGA) project were downloaded from the Genomic Data Commons Data Portal on 2020-06-05 [33]. Normalization and variance-stabilizing transformation (vst) of HTSeq count data were performed using DESeq2. Tumor and normal samples were split into different groups and only one sample per donor was kept in each group.

The TCGA LUAD exome mutation spectra were downloaded from Synapse [34] and decomposed into COSMIC v3 signatures SBS1, SBS2, SBS4, SBS5, SBS13, SBS40, and SBS45 using the quadratic programming (QP) approach available in the R package SignatureEstimation [35]. Only signatures predominantly active in lung cancer (signatures that were present in at least 5% of samples and were responsible for at least 1% of mutations) were considered based on the initial sample decomposition provided by Alexandrov et al. [2, 36]. Signature SBS45 is likely a sequencing artifact so it was omitted from further analyses presented in this study. The same log transformation used in BRCA analysis was performed on signature exposure data as well.

We analyzed 466 tumor samples that had both gene expression and mutational signature exposure data available. We analyzed 2433 genes belonging to the DNA metabolic process and immune system process in GO terms (genes that are not expressed in at least 10% of the samples were omitted). The gene expression and mutational signature exposure data were combined to form an input data matrix. All the data is available at the Github site https://github.com/ncbi/GeneSigNet [37].

The numbers of recovered edges and average degrees for each of the two cancer networks are summarized in Additional file 1: Table S1.

## Results

### Performance evaluation for GeneSigNet

#### Evaluation of the method on simulated data

GeneSigNet infers a directed network representing information flow between genes’ expression and signatures’ exposure. We compared the performance of GeneSigNet to four competing approaches which, similarly to GeneSigNet, strive to predict influence flow between network nodes. We compare GeneSigNet to the following key approaches: (1) the independent component analysis-based method LiNGAM [19], (2) the partial correlation-based heuristic GeneNet [20] previously proposed to discover the causal structure in high-dimensional genomic data [21], (3) the Sparse Partial Correlation Selection (SPCS) approach [21] and (4) ElasticNet [38]. Additionally, we included (5) the regression tree-based approach used by GENIE3 method [39] for the inference of Gene Regulatory Networks (GRN)  [39, 40, 41]. We note that the task of GRN inference is different from inferring influence graphs considered in this study. However, since the method produces a fully connected directed weighted graph we used a procedure that reduces bidirectional edges to oriented edges by retaining the heavier edge in the pair. Since GENIE3 is the winning gene expression-based GRN reconstruction method [42], this allowed us to test if a basic adaptation of a GRN reconstruction method can provide a competitive solution to our problem.

To generate data, we implemented the data simulation schema provided in LiNGAM [19]. This approach starts with the construction of a lower triangular weight matrix representing the directed weighted interactions among the nodes (random variables) in a directed acyclic graph (DAG). This matrix is then used as the representation of the dependency patterns in the generation of the data set (for detailed description of the data generation, see Additional file 1: Section S1.5). We simulated a set for 100 random variables (nodes) with 1000 samples from multivariate distributions. The weight matrix used in the simulation contains 364 directed edges.

**Fig. 4:**
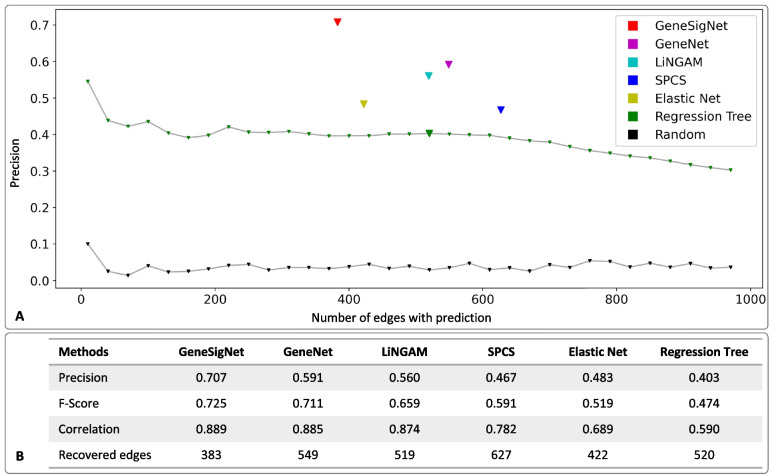
Evaluation of the results on simulated data. In **A**, the horizontal axis denotes the number of selected edges in the prediction. The vertical axis denotes the precision of the compared methods. In **B**, we report comparisons based on F-score, ability to reproduce the influence weight values used to generate the simulation data (correlation), and the number of the inferred edges

The performances of the methods are summarized in Fig. 4. GeneSigNet, GeneNet, LiNGAM, SPCS and ElasticNet construct a single network that is the output of the method-specific optimization procedure, thus their performances are represented by single points in Fig. 4A. The outcome of the regression tree approach can be defined differently depending on edge-weight cut-offs and its performance is thus represented by a set of points for various cut-offs. The results were similar for all cut-offs and for the remaining measures we report a representative result corresponding to 520 edges (big green triangle in Fig. 4A). GeneSigNet had the highest precision in predicting the direction of the influence. The F-score (the harmonic mean of precision and recall), was also the highest for GeneSigNet although only slightly better than the second best method, GeneNet. In addition, we also evaluated the methods on their ability to recover the values of the lower triangular matrix used to simulate the data. This measure was proposed in [19] and measures the correlation between edge weights used for the simulation and weights recovered by the algorithm. Despite inferring a smaller number of edges the GeneSigNet’ ability to reconstruct the input matrix was equal or better relative to other methods, suggesting that GeneSigNet indeed captured dominating influences using a smaller number of edges.

#### Evaluation of the directionality inference on real cancer data

**Fig. 5:**
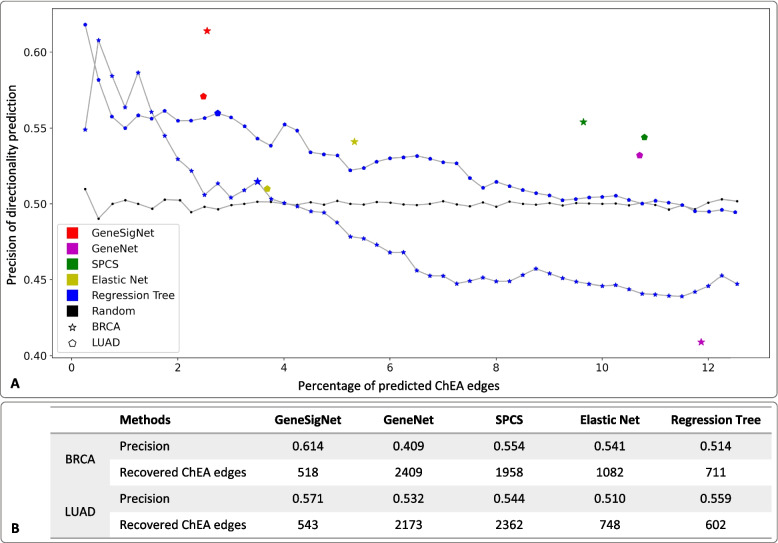
Correctness evaluation of directionality inference on real data sets. In **A**, the horizontal axis denotes the percent of ChEA edges for which a method made a prediction (out of all ChEA edges recovered by the method) whereas the vertical axis denotes the fraction of correctly predicted directions for these edges. In **B**, we provide the numerical values of the results visualized in **A**

We also compared the performance of the methods on breast cancer (BRCA) and lung cancer (LUAD) data sets (for details, see the “Materials” section). The LiNGAM method was excluded from this evaluation, since it cannot deal with large genome-wide datasets. In this setting the true direction of most of the inferred edges is, unfortunately, unknown. Therefore, we leverage the fact that if a method infers a relation between a transcription factor (TF) and a target gene (TG) then the edge should be directed from TF to TG (under the assumption that TG is not a TF). Leveraging this principle, we evaluated the precision of the directionality inference utilising the ChEA database containing directed protein-DNA interactions [27]. Specifically, for each method, we used the set of ChEA edges that overlap with the edges inferred by the method. Since the number of edges overlapping with ChEA set was different in each experiment, we used the percentage of overlap as the reference to ensure fairness of the comparison (horizontal axis in Fig. 5). GeneSigNet had the highest precision. Surprisingly, GeneNet and the regression tree methods resulted in worse than random performance in the evaluation on the BRCA data. Unfortunately, in the case of real data, the weights of the reference edges are unknown so we do not have a measure corresponding to the correlation function used on simulated data.

Overall the relations between the methods’ performances observed in this evaluation were very similar to the trends observed on simulated data supporting the choice of our network reconstruction strategy.

Finally, we tested the robustness of our new partial higher moment strategy used in the second step of GeneSigNet, by performing a bootstrap sampling which is described in Additional file 1: Section S1.3. The approach was highly reproducible as summarized in Additional file 1: Section S1.3 and Fig. S1. GeneSigNet compared favorably to the competing approaches.

The GeneSigNet method was implemented in python, and installable package, source codes and the data sets used for and generated during this study are available at the Github site https://github.com/ncbi/GeneSigNet [37].

### Analysis of the relations between mutational signatures and molecular pathways in breast cancer

We utilized breast cancer (BRCA) data collection obtained from ICGC which includes 266 cancer samples providing both whole-genome sequencing data and gene expression data (for details, see the “Materials” section: Breast cancer data). The breast cancer genomes harbor mutations mainly contributed by 6 COSMIC mutational signatures — SBS1, 2, 3, 5, 8, and 13. We further refined the mutational signatures based on mutation density and sample correlations. The mutations in BRCA are characterized by occurrences of short highly mutated regions whose origin is believed to be different compared to sparse mutations [8, 9, 43, 44, 45]. The information available from whole-genome sequencing allows for distinguishing these two types of mutation patterns and to treat such dense and sparse mutation regions differently. The post-processing of mutational signatures resulted in 6 signature groups that we use for subsequent analysis to construct the GSN – SBS1, APOBEC-C (clustered SBS2 and SBS13 corresponding to APOBEC hypermutation), APOBEC-D (SBS2 corresponding to disperse APOBEC mutations), DSB (SBS3 and clustered SBS8), SBS5, and SBS8D (dispersed SBS8). In addition to gene expressions and exposures of mutational signatures, we included a node indicating the binary status of homologous recombination deficiency (HRD) as it is assumed to lead to specific patterns of mutational signatures in BRCA [32]. We applied GeneSigNet to construct a GSN for genes, mutational signatures, and HRD status, and to find relations between these features.

#### Consistency of GeneSigNet results with current knowledge

**Fig. 6:**
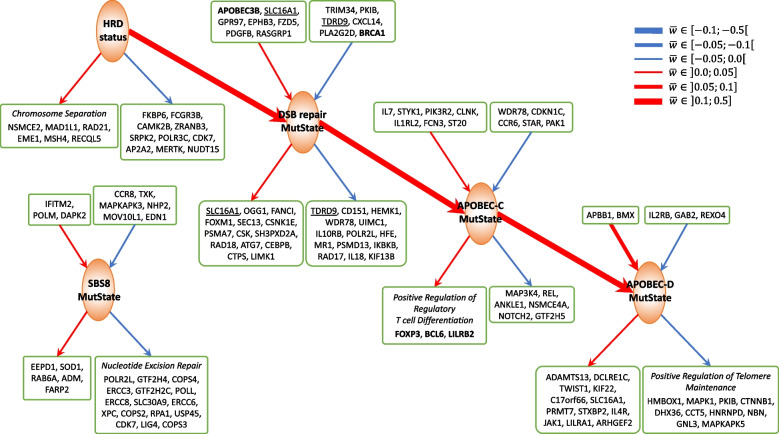
Subnetworks of GSN for BRCA centered on (induced by) MutStates associated with Homologous Recombination, APOBEC and SBS8. Edge and node colors are as in Fig. 1. In boxes, there are the names of the genes adjacent to a given MutState with edge weight cut-off ($|w_{ij}|\ge 0.01$). The genes in bold are discussed in more detail in the text and the genes having bidirected interactions with MutStates are underlined. If the adjacent genes are enriched with specific GO pathways ($q-value<0.01$) then only the pathway genes are provided in the box. The HRD status dominantly contributes to the mutation strength in the DSB repair MutState. Increased DSB exposure leads to an increase of the exposures of APOBEC-C and (indirectly) APOBEC-D MutStates. SBS8 mutations are linked to the deficiency of nucleotide excision repair. The thickness of the arrows represents the edge weight (average edge weight if multiple genes are in a box). An extended subnetwork including all MutStates and an extended list of genes, and GO-terms are provided in Additional file 1: Fig. S3, Additional file 2: Table S2, and Additional file 3: Table S4

Many relations uncovered with GeneSigNet are consistent with our current knowledge on mutational signatures, confirming the validity of our method. In particular, it is well appreciated that homologous recombination (HR) plays an important role in the double-strand break (DSB) repair mechanism and that HR deficiency is associated with the DSB signature [46]. Indeed, our network correctly predicted a strong positive influence from HRD status to the DSB signature (Fig. 6). In addition, GeneSigNet identified the known negative impact of BRCA1 expression on the DSB signature which is also consistent with the role of BRCA1 in HRD [46]. Furthermore, GeneSigNet captured the impact of HRD on chromosome separation, reflecting the role of homologous recombination in maintaining genomic stability [47, 48], and identified the association of APOBEC-D with telomere maintenance, consistent with the well recognized role of APOBEC mutagenesis in replication [49, 50].

Interestingly, our method linked SBS8 to the nucleotide excision repair (NER) pathway (Fig. 6). The etiology of this signature has remained unknown until a recent experimental study linked it to the NER pathway as well [25]. This demonstrates the power of the GeneSigNet method to uncover non-obvious relationships.

#### Untangling the interactions between APOBEC and DSB processes

Previous studies speculated that APOBEC related mutational signatures can arise in multiple different scenarios. First, double-strand breaks (DSB) created by the homologous recombination deficiency (HRD) provide mutational opportunities for APOBEC enzymes to act on the ssDNA regions, resulting in clustered APOBEC mutations [45, 51, 52]. In another scenario, a recent study attributed APOBEC-mediated hypermutations to the normal activity of mismatch repair which also involves creating ssDNA regions, generating “fog” APOBEC mutations [44]. The complex interplay between APOBEC activities and other DNA repair mechanisms is yet to be elucidated.

Focusing on the interactions of APOBEC signatures with the other MutStates and genes, we observe that GeneSigNet supports a positive influence of the DSB on APOBEC-C MutState, consistent with the assumption that double-strand breaks provide an opportunity for APOBEC mutations. Additionally, our analysis reveals that the expression level of the APOBEC3B enzyme is associated with the strength of the DSB signature. Indeed, a previous study proposed that APOBEC3 proteins are recruited to DSB sites to participate in the DSB repair process [14]. Thus, DSB contributes to an increase in APOBEC-C strength by two different mechanisms: (i) increased mutation opportunity due to ssDNA created by DSB and (ii) increased mutation probability due to increased APOBEC3B expression. Note that increased APOBEC expression would also increase APOBEC mutations in the “fog” regions proposed in [44].

On the other hand, the activity of APOBEC-D is positively influenced by APOBEC-C activity, without direct relation to DSB. In fact, GeneSigNet inferred a negative influence from HR status to APOBEC-D MutState, confirming that different mutagenic processes are involved in clustered and dispersed APOBEC mutations (Fig. 6).

#### APOBEC hypermutation activates regulatory T cells — implications for immunotherapy

Interestingly, GO enrichment analysis of the genes associated with APOBEC mutational signatures (genes influenced by APOBEC-C MutState) revealed significant enrichment in positive regulation of regulatory T cells (Tregs) differentiation (Fig. 6). Tregs, a subtype of T cells that suppress the immune response, are important for maintaining cell homeostasis and self-tolerance but can also interfere with anti-tumor immune response [53]. The top three genes (FOXP3, BCL6, and LILRB2) positively influenced by APOBEC-C signature are all related to such inhibitory mechanism to immune response [54, 55, 56]. FOXP3 is a transcriptional regulator playing a crucial role in the inhibitory function of Tregs. BCL6 is also essential for the stability of Tregs that promotes tumor growth. LILRB2 is a receptor for class I MHC antigens and is involved in the down-regulation of the immune response and the development of immune tolerance.

Our results help to understand a complicated role of APOBEC mutagenesis holds for immunotherapy. For example, patients with cancers displaying a high mutation burden are likely to produce tumor-associated neoantigens (mutated peptides presented at their surface) allowing them to benefit from immunotherapy [57]. In particular, the APOBEC mutational signature was identified as a potential predictive marker for immunotherapy response in some cancers [58, 59]. Yet, cells carrying a high mutation burden often develop mechanisms of immune tolerance involving activation of Tregs to protect themselves from the destruction [60, 61]. Consequently, observed increased number of Tregs in response to high APOBEC mutations may lead to resistance to immune checkpoint inhibitors [62, 63]. Thus, our finding suggests that a combined strategy targeting Tregs in addition to immune checkpoint inhibitors would be most beneficial for a better outcome in APOBEC hypermutated breast cancer tumors.

### Analysis of the relations between mutational signatures and molecular pathways in Lung Adenocarcinoma

We next analyzed lung adenocarcinoma (LUAD) data using 466 cancer samples from the TCGA project. The exposure levels of 6 COSMIC mutational signatures (SBS1, 2, 4, 5, 13, and 40) present in the exome sequencing data were integrated with the RNAseq expression data of 2433 genes belonging to the DNA metabolic and immune system processes in Gene Ontology terms to uncover influence between signatures and genes (for details, see the “Materials” section: Lung adenocarcinoma data).

#### GeneSigNet uncovers immune response due to smoking

**Fig. 7:**
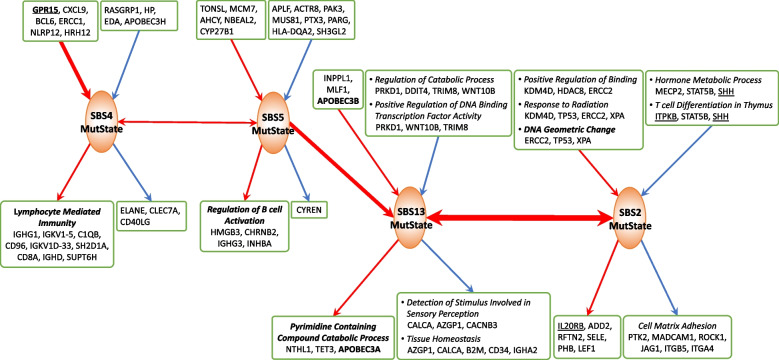
Subnetwork of LUAD GSN centered on MutStates known to be related to smoking and APOBEC. The meaning of colors and boxes is the same as in Figs. 1 and 6. An extended subnetwork including all MutStates and an extended list of genes, and GO-terms are provided in Additional file 1: Fig. S4, Additional file 2: Table S3, and Additional file 3: Table S5

Two prominent mutational signatures in LUAD, SBS4 and SBS5, are assumed to result from exogenous causes [8]. SBS4 is associated specifically with exposure to cigarette smoking in lungs. SBS5 is known to accompany the smoking signature but it is also present in many other cancer types. Previous studies suggested that cigarette smoking stimulates an inflammatory response [10]. Consistent with these findings, the genes identified by GeneSigNet as influenced by SBS4 and SBS5 MutStates are indeed enriched with immune response genes (Fig. 7).

GeneSigNet also identified the influence of the signatures SBS4 and SBS5 on two APOBEC signatures — SBS2 and SBS13. The APOBEC signatures are associated with immune response and this relationship is consistent with the previously proposed immune activation due to smoking exposure [64]. In addition, GeneSigNet correctly captured the association of SBS13 (consequently SBS2) with the expressions of APOBEC3B and APOBEC3A enzymes, and also identified the association of SBS13 with pyrimidine related catabolic processes, potentially reflecting the fact that SBS13 involves a pyrimidine to pyrimidine mutation (Fig. 7).

**Fig. 8:**
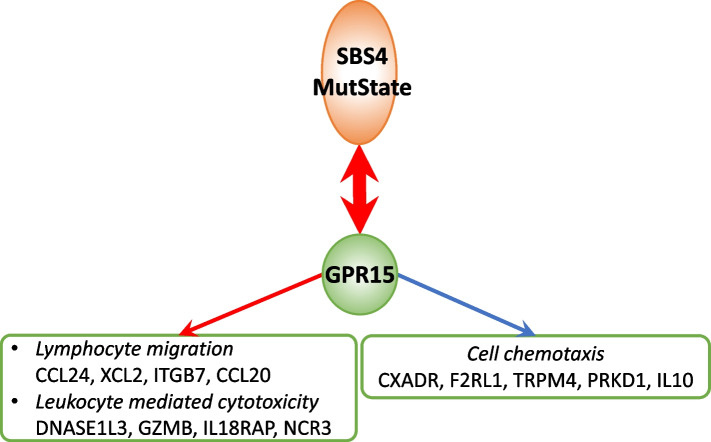
Subnetwork of Lung Adenocarcinoma GSN centered on the node representing GPR15 gene. Expression of the GPR15 gene contributes to the activation of immune responses. The meaning of edge and node colors, and boxes is the same as in Figs. 1 and 6. An extended subnetwork including all MutStates and an extended list of genes is provided as Additional file 1: Fig. S4

Finally, tobacco smoking is known to induce GPR15-expressing T cells; although the exact role of GPR15 in response to smoking is yet to be elucidated [65]. Therefore, we investigated whether the results of GeneSigNet provide additional insights into this relation. Consistently with previous studies, GeneSigNet inferred a strong association between GPR15 and SBS4 without resolving the direction (see also Additional file 1: Fig. S4). Next, we analyzed the influence that GPR15 has on other nodes of the GSN network. The results of GeneSigNet suggest that GPR15 is involved in the negative regulation of several genes related to chemotaxis, including IL10, a cytokine with potent anti-inflammatory properties, and has a positive impact on lymphocyte migration and leukocyte mediated cytotoxicity (Fig. 8).

#### GeneSigNet points to the role of DNA geometric changes for APOBEC signature SBS2

As discussed earlier, APOBEC can only act on single-stranded DNA (ssDNA). Interestingly, one of the GO terms associated with SBS2 MutState identified by GeneSigNet is DNA geometric change (Fig. 7). DNA geometric changes are local changes of DNA conformation such as bulky DNA adducts (a type of DNA damage due to exposure to cigarette smoke) or DNA secondary structures such as Z-DNA, cruciforms, or quadruplexes. Indeed, these structures often involve the formation of ssDNA regions which, in turn, provide mutation opportunities for APOBEC enzymes [66, 67, 68]. The formation of DNA secondary structures is often associated with DNA supercoiling — a form of DNA stress that is resolved by Topoisomerase 1 (TOP1). Interestingly, GeneSigNet identified a negative influence of TOP1 expression on one of the genes (XPA) contributing to this GO term. This suggests a relation between DNA stress mediated by TOP1 and APOBEC activity.

## Discussion

Elucidating the nature of mutagenic processes and their interactions with cellular processes is of fundamental importance for understating cancer etiology and guiding cancer therapy. Here, we propose GeneSigNet, a new network-based approach that infers the relation between gene expression and the strength of mutation patterns (signature exposures) allowing us to uncover the relations between signatures and processes involved in DNA repair and immune response among other cellular processes. Recognizing the limitations of the previous approaches, GeneSigNet relies on a construction of a sparse directed network. For each node (gene or MutState), it selects a sparse set of incoming edges representing dominating incoming effects so that their combination explains the activity of the node. Aiming to capture the most dominant influences, the method utilizes sparse partial correlation coefficients.

In this study we focused on the relations that can be inferred based on genes’ expressions and exposures of mutational signatures. It is possible to include other features as well. We leveraged this flexibility of our method to include for breast cancer germline perturbations that are known to predispose homologous recombination deficiency (HD).

The construction of a sparse network utilized by our approach has both advantages and disadvantages. Sparse networks are easier to interpret and more likely to suggest mechanistic insights. However, by restricting edge set, they might lose some information. In our previous studies [8, 69] we have developed a clustering based approach that leverages gene co-expression. These two approaches illustrate the trade-off between a gene-level analysis which has the potential for more mechanistic explanation (e.g., association of Tregs with APOBEC we have identified in this paper) while potentially losing some information and a cluster-level analysis which is empowered by the correlations between expression of genes but provides GO-term interpretability only. In future studies, it could be beneficial to consider a hierarchical representation that can work on many levels of granularity simultaneously, combining the strengths of both approaches.

For the biological results, overall the relations discovered by GeneSigNet are consistent with the current knowledge, boosting the confidence in the method’s applicability. In addition, GeneSigNet provided several new biological insights concerning the relation between mutagenic processes and other cellular processes. For example, the uncovered relation between APOBEC hypermutation and activation of regulatory T cell can have an important implication in immunotherapy.

## Conclusions

In this work, we report a computational method, named GeneSigNet, for uncovering potentially causal relations between gene expression and mutagenic processes represented by mutational signatures. The main idea of the approach is to construct an influence network representing dependency flow between genes and mutational signatures. We opted to infer a sparse network in order to focus on the strongest trends that are more likely to suggest mechanistic explanations. We note that focusing on a sparse set of edges reduces the power of GO enrichment analysis and requires more specific biological knowledge for interpreting the results. Yet, this potential disadvantage is compensated by the compelling mechanistic insights provided by the method.

## Supplementary information

**Additional file 1.** Supplemental Information includes Sections S1, S1.1, S1.2, S1.3, S1.4, and S1.5: detailed descriptions of the GeneSigNet, and data simulation schema for validation, Table S1: Supplemental table providing the topological information on the inferred networks, Figs. S1, S2, S3, and S4: Supplemental figures for visualizing the performance of the GeneSigNet method and the extended subnetworks covering the mutational signatures and their direct neighbors. (PDF)

**Additional file 2.** Tables S2 (BRCA), and S3 (LUAD) : Supplemental tables providing the genes enriched in up and down streams of mutational signatures and their interaction weights in the gene signature networks of BRCA and LUAD. (Excel)

**Additional file 3.** Tables S4 (BRCA) and S5 (LUAD): Supplemental tables providing the GO terms enriched for causal and affected genes of mutational signatures in the gene signature networks of BRCA and LUAD. (Excel)

## Data Availability

The GeneSigNet method was implemented in python, and installable package, source codes and the data sets used for and generated during this study are available at the Github site https://github.com/ncbi/GeneSigNet [37].

## References

[1] Alexandrov LB, Nik-Zainal S, Wedge DC, Campbell PJ, Stratton MR (2013). Deciphering signatures of mutational processes operative in human cancer. Cell Rep..

[2] Alexandrov LB, Kim J, Haradhvala NJ, Huang MN, Tian Ng AW, Wu Y (2020). The repertoire of mutational signatures in human cancer. Nature..

[3] Kim YA, Leiserson MDM, Moorjani P, Sharan R, Wojtowicz D, Przytycka TM (2021). Mutational Signatures: From Methods to Mechanisms. Annu Rev Biomed Data Sci..

[4] Viel A, Bruselles A, Meccia E, Fornasarig M, Quaia M, Canzonieri V (2017). A Specific Mutational Signature Associated with DNA 8-Oxoguanine Persistence in MUTYH-defective Colorectal Cancer. EBioMedicine..

[5] Kim J, Mouw KW, Polak P, Braunstein LZ, Kamburov A, Kwiatkowski DJ (2016). Somatic ERCC2 mutations are associated with a distinct genomic signature in urothelial tumors. Nat Genet..

[6] Zou X, Owusu M, Harris R, Jackson SP, Loizou JI, Nik-Zainal S (2018). Validating the concept of mutational signatures with isogenic cell models. Nat Commun..

[7] Volinia S, Druck T, Paisie CA, Schrock MS, Huebner K (2017). The ubiquitous ‘cancer mutational signature’ 5 occurs specifically in cancers with deleted FHIT alleles. Oncotarget..

[8] Kim YA, Wojtowicz D, Sarto Basso R, Sason I, Robinson W, Hochbaum DS (2020). Network-based approaches elucidate differences within APOBEC and clock-like signatures in breast cancer. Genome Med..

[9] Wojtowicz D, Sason I, Huang X, Kim YA, Leiserson MDM, Przytycka TM (2019). Hidden Markov models lead to higher resolution maps of mutation signature activity in cancer. Genome Med..

[10] Alexandrov LB, Ju YS, Haase K, Van Loo P, Martincorena I, Nik-Zainal S (2016). Mutational signatures associated with tobacco smoking in human cancer. Science..

[11] Wong JKL, ller C, Schulze M, Hlevnjak M, Elgaafary S, Lichter P (2022). Association of mutation signature effectuating processes with mutation hotspots in driver genes and non-coding regions. Nat Commun..

[12] Temko D, Tomlinson IPM, Severini S, Schuster-Böckler B, Graham TA (2018). The effects of mutational processes and selection on driver mutations across cancer types. Nat Commun..

[13] Cook JH, Melloni GEM, Gulhan DC, Park PJ, Haigis KM (2021). The origins and genetic interactions of KRAS mutations are allele- and tissue-specific. Nat Commun..

[14] Nowarski R, Kotler M (2013). APOBEC3 cytidine deaminases in double-strand DNA break repair and cancer promotion. Cancer Res..

[15] Silverbush D, Sharan R (2014). Network orientation via shortest paths. Bioinformatics..

[16] Gitter A, Klein-Seetharaman J, Gupta A, Bar-Joseph Z (2011). Discovering pathways by orienting edges in protein interaction networks. Nucleic Acids Res..

[17] Vinayagam A, Stelzl U, Foulle R, Plassmann S, Zenkner M, Timm J (2011). A directed protein interaction network for investigating intracellular signal transduction. Sci Signal..

[18] Silverbush D, Sharan R (2019). A systematic approach to orient the human protein-protein interaction network. Nat Commun..

[19] Shimizu S, Hoyer P, Hyvärinen A, Kerminen A, Jordan M. A linear non-Gaussian acyclic model for causal discovery. J Mach Learn Res. 2006;7(10):2003–30.

[20] Opgen-Rhein R, Strimmer K (2007). From correlation to causation networks: a simple approximate learning algorithm and its application to high-dimensional plant gene expression data. BMC Syst Biol..

[21] Amgalan B, Lee H (2015). DEOD: uncovering dominant effects of cancer-driver genes based on a partial covariance selection method. Bioinformatics..

[22] Dodge Y, Yadegari I (2010). On direction of dependence. Metrika..

[23] Moretton A, Loizou JI. Interplay between Cellular Metabolism and the DNA Damage Response in Cancer. Cancers (Basel). 2020;12(8):2051. 10.3390/cancers12082051 PMC7463900 32722390

[24] Nakad R, Schumacher B (2016). DNA Damage Response and Immune Defense: Links and Mechanisms. Front Genet..

[25] Jager M, Blokzijl F, Kuijk E, Bertl J, Vougioukalaki M, Janssen R (2019). Deficiency of nucleotide excision repair is associated with mutational signature observed in cancer. Genome Res..

[26] Fujikoshi Y, Ulyanov VV. Shimizu R. Multivariate statistics: High-dimensional and large-sample approximations. Book. 2011;187–210.

[27] Lachmann A, Xu H, Krishnan J, Berger SI, Mazloom AR, Ma’ayan A (2010). ChEA: transcription factor regulation inferred from integrating genome-wide ChIP-X experiments. Bioinformatics..

[28] Sinkhorn R, Knopp P (1967). Concerning nonnegative matrices and doubly stochastic matrices. Pac J Math..

[29] Nik-Zainal S, Davies H, Staaf J, Ramakrishna M, Glodzik D, Zou X (2019). Author Correction: Landscape of somatic mutations in 560 breast cancer whole-genome sequences. Nature..

[30] ICGC data portal. https://dcc.icgc.org. Accessed on 1 May 2020.

[31] COSMIC v2 signature. https://cancer.sanger.ac.uk/cosmic/signature. Accessed on 1 May 2020.

[32] Davies H, Glodzik D, Morganella S, Yates LR, Staaf J, Zou X (2017). HRDetect is a predictor of BRCA1 and BRCA2 deficiency based on mutational signatures. Nat Med..

[33] Data Commons Data Portal. https://portal.gdc.cancer.gov. Accessed on 1 May 2020.

[34] Synapse data portal. https://www.synapse.org/#!Synapse:syn11801889. Accessed on 1 May 2020.

[35] Huang X, Wojtowicz D, Przytycka TM (2018). Detecting presence of mutational signatures in cancer with confidence. Bioinformatics..

[36] Synapse data portal. https://www.synapse.org/#!Synapse:syn11804065. Accessed on 1 May 2020.

[37] Amgalan A. A network-based approach to infer causality flows among genes and mutational signatures. Github. 2022. https://github.com/ncbi/GeneSigNet. Accessed on 12 Nov 2022.

[38] Zou H, Hastie T (2005). Regularization and variable selection via the elastic net. J R Stat Soc Ser B (Stat Methodol)..

[39] Huynh-Thu VA, Irrthum A, Wehenkel L, Geurts P. Inferring regulatory networks from expression data using tree-based methods. PLoS ONE. 2010;5(9): e12776. 10.1371/journal.pone.0012776 PMC2946910 20927193

[40] Kang Y, Thieffry D, Cantini L (2021). Evaluating the Reproducibility of Single-Cell Gene Regulatory Network Inference Algorithms. Front Genet..

[41] Huynh-Thu VA, Geurts P (2018). dynGENIE3: dynamical GENIE3 for the inference of gene networks from time series expression data. Sci Rep..

[42] Marbach D, Costello JC, Küffner R, Vega NM, Prill RJ, Camacho DM, et al. "Wisdom of crowds for robust gene network inference." Nat Methods. 2012; 9(8):796–804. 10.1038/nmeth.2016 PMC3512113 22796662

[43] Supek F, Lehner B (2017). Clustered Mutation Signatures Reveal that Error-Prone DNA Repair Targets Mutations to Active Genes. Cell..

[44] Mas-Ponte D, Supek F (2020). DNA mismatch repair promotes APOBEC3-mediated diffuse hypermutation in human cancers. Nat Genet..

[45] Taylor BJ, Nik-Zainal S, Wu YL, Stebbings LA, Raine K, Campbell PJ (2013). DNA deaminases induce break-associated mutation showers with implication of APOBEC3B and 3A in breast cancer kataegis. Elife..

[46] Prakash R, Zhang Y, Feng W, Jasin M (2015). Homologous recombination and human health: the roles of BRCA1, BRCA2, and associated proteins. Cold Spring Harb Perspect Biol..

[47] Moynahan ME, Jasin M (2010). Mitotic homologous recombination maintains genomic stability and suppresses tumorigenesis. Nat Rev Mol Cell Biol..

[48] Li X, Heyer WD (2008). Homologous recombination in DNA repair and DNA damage tolerance. Cell Res..

[49] Seplyarskiy VB, Soldatov RA, Popadin KY, Antonarakis SE, Bazykin GA, Nikolaev SI (2016). APOBEC-induced mutations in human cancers are strongly enriched on the lagging DNA strand during replication. Genome Res..

[50] Kanu N, Cerone MA, Goh G, Zalmas LP, Bartkova J, Dietzen M (2016). DNA replication stress mediates APOBEC3 family mutagenesis in breast cancer. Genome Biol..

[51] Sakofsky CJ, Saini N, Klimczak LJ, Chan K, Malc EP, Mieczkowski PA (2019). Repair of multiple simultaneous double-strand breaks causes bursts of genome-wide clustered hypermutation. PLoS Biol..

[52] Chan K, Gordenin DA (2015). Clusters of Multiple Mutations: Incidence and Molecular Mechanisms. Annu Rev Genet..

[53] Facciabene A, Motz GT, Coukos G (2012). T-regulatory cells: key players in tumor immune escape and angiogenesis. Cancer Res..

[54] Rudensky AY (2011). Regulatory T cells and Foxp3. Immunol Rev..

[55] Chung Y, Tanaka S, Chu F, Nurieva RI, Martinez GJ, Rawal S (2011). Follicular regulatory T cells expressing Foxp3 and Bcl-6 suppress germinal center reactions. Nat Med..

[56] Chen HM, van der Touw W, Wang YS, Kang K, Mai S, Zhang J (2018). Blocking immunoinhibitory receptor LILRB2 reprograms tumor-associated myeloid cells and promotes antitumor immunity. J Clin Invest..

[57] Sholl LM, Hirsch FR, Hwang D, Botling J, Lopez-Rios F, Bubendorf L (2020). The Promises and Challenges of Tumor Mutation Burden as an Immunotherapy Biomarker: A Perspective from the International Association for the Study of Lung Cancer Pathology Committee. J Thorac Oncol..

[58] Wang S, Jia M, He Z, Liu XS (2018). APOBEC3B and APOBEC mutational signature as potential predictive markers for immunotherapy response in non-small cell lung cancer. Oncogene..

[59] Faden DL, Ding F, Lin Y, Zhai S, Kuo F, Chan TA (2019). APOBEC mutagenesis is tightly linked to the immune landscape and immunotherapy biomarkers in head and neck squamous cell carcinoma. Oral Oncol..

[60] Chan TA, Yarchoan M, Jaffee E, Swanton C, Quezada SA, Stenzinger A (2019). Development of tumor mutation burden as an immunotherapy biomarker: utility for the oncology clinic. Ann Oncol..

[61] Venkatesan S, Rosenthal R, Kanu N, McGranahan N, Bartek J, Quezada SA (2018). Perspective: APOBEC mutagenesis in drug resistance and immune escape in HIV and cancer evolution. Ann Oncol..

[62] Saleh R, Elkord E (2019). Treg-mediated acquired resistance to immune checkpoint inhibitors. Cancer Lett..

[63] Principe DR, Chiec L, Mohindra NA, Munshi HG (2021). Regulatory T-Cells as an Emerging Barrier to Immune Checkpoint Inhibition in Lung Cancer. Front Oncol..

[64] Patriarca PA, Foege WH, Swartz TA (1993). Progress in polio eradication. Lancet..

[65] Kõks S, Kõks G (2017). Activation of GPR15 and its involvement in the biological effects of smoking. Exp Biol Med (Maywood)..

[66] Kouzine F, Wojtowicz D, Baranello L, Yamane A, Nelson S, Resch W (2017). Permanganate/S1 Nuclease Footprinting Reveals Non-B DNA Structures with Regulatory Potential across a Mammalian Genome. Cell Syst..

[67] Zou X, Morganella S, Glodzik D, Davies H, Li Y, Stratton MR (2017). Short inverted repeats contribute to localized mutability in human somatic cells. Nucleic Acids Res..

[68] Guiblet WM, Cremona MA, Harris RS, Chen D, Eckert KA, Chiaromonte F (2021). Non-B DNA: a major contributor to small- and large-scale variation in nucleotide substitution frequencies across the genome. Nucleic Acids Res..

[69] Kim YA, Hodzic E, Amgalan B, Saslafsky A, Wojtowicz D, Przytycka TM. Mutational Signatures as Sensors of Environmental Exposures: Analysis of Smoking-Induced Lung Tissue Remodeling. Biomolecules. 2022;12(10):1384–13. 10.3390/biom12101384 PMC9599238 36291592

