# Supplementary material for: Influence network model uncovers relations between biological processes and mutational signatures
Source: Genome Med. 2023 Mar 6;15:15. doi: 10.1186/s13073-023-01162-x (PMC9987115; doi:10.1186/s13073-023-01162-x)
Supplement: Supplementary file 1 — Additional file 1. Supplemental Information includes Sections S1, S1.1, S1.2, S1.3, S1.4, and S1.5: detailed descriptions of the GeneSigNet, and data simulation schema for validation, Table S1: Supplemental table providing the topological information on the inferred networks, Figs. S1, S2, S3, and S4: Supplemental figures for visualizing the performance of the GeneSigNet method and the extended subnetworks covering the mutational signatures and their direct neighbors. (PDF) [file 13073_2023_1162_MOESM1_ESM.pdf]

Supplementary Information

S1 Supplementary Methods

GeneSigNet constructs a sparse network representing the directed dependencies among nodes in a Gene-Signature Network (GSN). The nodes corresponding to genes and MutStates (mutational signatures) are considered random variables and the activities of the variables are denoted as expressions of genes and exposures of mutational signatures across patients (samples). Given an input matrix describing the activities of variables, our network construction method consists of two complementary steps: (i) a sparse partial correlation technique (SPCS) to obtain an initial weighted-directed network by estimating statistical dependencies (edges) between variables (nodes) and (ii) a partial higher moment strategy to refine the initial network by orienting bidirectional edge. A high-level description of GeneSigNet is provided in Section 2.1 in the manuscript, and the detailed description and related literature are presented below.

S1.1 A sparse partial correlation selection (SPCS)

In the proposed model, the network inference algorithm does not distinguish between the two types of nodes (genes and MutStates) and infers dependencies between variables based on their observed activities. In this setting, correlation networks are widely used to explore and visualize dependencies in high-dimensional data. However, without assuming prior knowledge, an ordinary correlation itself provides no means to distinguish between influence and affected factors in underlying causal processes (Opgen-Rhein and Strimmer 2007).

Bayesian networks can be used to infer causal relations of nodes representing their local conditional dependencies via a directed acyclic graph (DAG) (S. Shimizu et al. 2006; Opgen-Rhein and Strimmer 2007; Friedman et al. 2000; Tsagris 2019; Scutari, Vitolo, and Tucker 2019). Alternative to constructing a DAG, directed partial correlation (DPC) (Yuan, Li, and Windram 2011) and regression tree based GENIE3 (Huynh-Thu et al. 2010) methods have been proposed to uncover conditional dependencies in observed data. However, learning the structure of Bayesian networks from large data is computationally challenging (Scutari, Vitolo, and Tucker 2019) and the inferred networks are always acyclic and thus they do not support feedback loops (Jensen and Nielsen 2007). On the other hand, DPC and GENIE3 return a complete list of interactions with non-zero weights of connectivity strengths, hence generating fully-connected networks in which the choice of an optimal confidence threshold is left open. Other methods such as the sparse partial correlation estimation (SPACE) (Peng et al. 2009) and its extension (ESPASE) (Yu et al. 2017), consider a penalized regression approach to construct the gene regulatory network. Specifically, their joint loss function requires predetermined penalty weights to combine the regression losses over all the response variables. Although the two methods utilize sparse variable selections, both estimations provide symmetric weight matrices representing weighted-undirected networks.

In this work, we modeled directional dependencies as sparse partial correlation coefficients which are obtained by minimizing a least square error subject to the unit l_1 norm ball. Inspired by the theoretical foundations for approximating partial correlations (Box 1), SPCS selects the best combination of a small number of explanatory factors that, under the conditional dependency assumption, explains the activity of each node in the GSN (Fig. 2B in the manuscript).

Box 1: Partial correlations can be approximated by multi-regression coefficients

Consider the ordinary correlation ρ_{12} between two random variables v_1 and v_2. If v_1 and v_2 are correlated with $n-2$ other variables $v_3, v_4, \ldots, v_n$, we may regard ρ_{12} as a mixture of a direct correlation between v_1 and v_2 and an indirect portion due to the presence of other variables correlating with v_1 and v_2. The partial correlation measuring the direct portion of the total correlation can be represented as a correlation between v_1 and v_2 after removing effects due to other variables by a linear regression and the least square linear regression coefficients are proportional to the partial correlation coefficients (Fujikoshi, Ulyanov, and R. Shimizu 2011).

The entire network is represented as a weighted-directed graph, $\mathbb{G} = (\mathbb{V}, \mathbb{E})$, where a set of nodes $\mathbb{V}$ represents genes and MutStates, and a set of edges $\mathbb{E}$ represents the relationships among the nodes. As mentioned in Section 2.1 in the manuscript, the network inference algorithm does not distinguish between the two types of nodes and assumes the nodes as random variables.

Let $\mathbb{I}$ denote the index set of n variables representing the nodes in $\mathbb{V}$. The nodes have observational activities over samples, and a $p \times n$ matrix $X = \{x_{ij}\}$ represents the data consisting of expressions of m genes and exposures of $n-m$ mutational signatures across p samples (patients). Assuming the incoming effects on a given variable f from its dominating covariates, the observed value x_{if}, corresponding to i-th sample, can be approximated as the following affine combination

$$x_{if} \approx \sum_{k \in \mathbb{I} \setminus \{f\}} x_{ik} w_{kf} + w_{0f} \tag{S1}$$

where $\mathbb{I} \setminus \{f\}$ denotes the index set of all variables except for the response variable f, and $w_{*f} \in R^{n-1}$ denotes the weights of incoming effects on f from the other $n-1$ variables and w_{0f} denotes the intercept adjusting the fitness between the response variable and its prediction.

Thus, our goal is to find the minimum of the least square error function subject to a unit l_1 norm constraint on w_{*f} as following

$$\begin{aligned} \underset{w_{*f} \in R^{n-1}, w_{0f} \in R}{\text{minimize}} \quad & \sum_{i=1}^{p} \left(x_{if} - \sum_{k \in \mathbb{I} \setminus \{f\}} x_{ik} w_{kf} - w_{0f} \right)^2 \\ \text{subject to} \quad & \sum_{k \in \mathbb{I} \setminus \{f\}} |w_{kf}| \leq 1 \end{aligned} \tag{S2}$$

For a response node (affected) f, a vector $w_{*f} = (w_{1f}, w_{2f}, \ldots w_{f-1f}, w_{f+1f}, \ldots w_{nf})^T$, the solution to the problem in Equation (S2), represents the weights of incoming effects from the other $n-1$ nodes (influence factors) in the network. The l_1 norm constraint pushes the weights of insignificant effects towards zero and allows selecting only dominant influences on the given response variable. Hence, this regularization acts to avoid over-fitting issues. A non-zero w_{kf} ($k \neq f$), selected for the node f, denotes the partial correlation coefficient representing the potential effect of the node k on the node f. In this setting, focusing on the activity of every single node in the network, the optimization problem in Equation (S2) considers all possible combinations of incoming effects from the other $n-1$ nodes and selects the best combination with their optimal influence weights to explain the given response activity under conditional dependency assumption. Therefore, the directed relationship between nodes k and f is estimated in the presence of the other $n-2$ variables.

The major advantage of using l_1-norm regularization is that this approach provides a sparse solution which helps interpreting the estimated networks. In addition, it does not not require

statistical thresholding to infer such significant network connections. The l_1-norm regularization allows the model to select only strong connections while the weaker weights are shrunk to zero. As a result the number of edges is reduced. This is not true for the l_2 norm regularization which makes the makes the weights of less relevant edges smaller, but it does not set them exactly to zero.

S1.2 Solving SPCS model

An accurate solution of the problem in Equation (S2) is critical for the robust estimation of dependencies in the GSN. Although the least square error function is convex, the l_1 norm sparsity constraint is non-differentiable and derivative-based techniques such as Lagrange multipliers and Karush-Kuhn-Tucker (KKT) conditions are not directly applicable here due to the non-smoothness. Another attempt to resolve such an issue is to decompose the inequality of the l_1 norm into 2^n inequality constraints (Tibshirani 1996). However, biological networks are often large in scale and it is practically difficult to accurately minimize such a large scale objective function over the exponential number of constraints within a reasonable time. Thus, to approximate the non-smooth constrained optimization, we rewrite the initial formulation in Equation (S2) as an unconstrained form with a penalty term

$$\underset{w_{*f}\in R^{n-1}, w_{0f}\in R}{\text{minimize}} \quad \sum_{i=1}^{p}\left(x_{if} - \sum_{k\in\mathbb{I}\setminus\{f\}} x_{ik}w_{kf} - w_{0f}\right)^2 + \lambda_f \cdot \sum_{k\in\mathbb{I}\setminus\{f\}} |w_{kf}| \tag{S3}$$

where a tuning parameter λ_f controls the strength of the penalty term, chosen for the given variable f to provide a balance between the least square error term and the l_1 norm constraint in the formulation in Equation (S2). In general, describing the criterion to define a reasonable value of λ_f is not trivial, due to the incompleteness of information linked to biological relevance. In fact, the exact relationship between the radius of the l_1 norm ball in Equation (S2) and the tuning parameter λ_f in Equation (S3) is assumed to be data-dependent. Therefore, it is reasonable to use a data-driven strategy for choosing λ_f. Akaike information criterion (AIC) is a statistical technique that provides the relative quality of statistical models for a given data by combining the maximum likelihood estimation of fitness with the number of parameters for inference (Akaike 1974). The AIC is used in this work to decide the value of λ_f providing a solution with reasonable total incoming effect on f from its dominating factors.

S1.3 Partial higher moment strategy for influence direction

The solution to the problem in Equation (S2) may provide non-zero weights in both directions for some pairs of variables due to the presence of effects from confounding factors and noise in addition to their real dependencies. This uncertainty may require a complimentary analysis to decide the influence directions of the dependencies.

One way to decide direction of dependency is to perform perturbation experiments (Pearl 2019). However, optimization of experimental design to predict which combination of perturbations allows to discover influence flows in a given network topology is often challenging and costly (Chu et al. 2003). Hence, revealing influence directions by analyzing purely observational data has become a special focus of network biology (Glymour, Zhang, and Spirtes 2019). Under a confounder-free assumption, higher moment statistics (Dodge and Yadegari 2010) indicate influence direction between two dependent variables from purely observational data. Alternative to the directionality decision for bivariate distributions, a confounder model (Wiedermann and Sebastian 2020) was recently designed to assign influence directions for several factors under a standard dependency

assumption. By combining the key ideas of the two methods, we propose a partial higher moment strategy to propose the influence directions for the bidirected edges in the initial network. The idea is to generate a bivariate distribution for two correlated variables by removing confounding effects from their observed activities, and then decide the influence direction between them using higher moment statistics on the corresponding residual activities (Fig. 2C in the manuscript). Specifically, for two variables having effects on each other (bidirectional edge) in the initial GSN, we first calculate their residual activities by removing effects due to the presence of the other $n-2$ variables (Fujikoshi, Ulyanov, and R. Shimizu 2011). Upon the removal, the corresponding residuals are expected to follow a bivariate distribution and only the dependency between the focused pair remains in their residual activities. Thus, the influence variable can be distinguished from the affected by comparing the higher moments of the two residual distributions. Under the confounder-free assumption, the affected variable is closer to normality than the influence factor and the skewness and kurtosis are the partial higher moment statistics used to measure close-normality of the residual distributions.

Let x_{*j} and x_{*f} be j-th and f-th columns of the given data matrix X representing the observed activities of the variables j and f respectively. Then, the residual activities corresponding to the variables j and f can be obtained as follows

$$\begin{aligned} r_j &= x_{*j} - \sum_{k \in \mathbb{I} \setminus \{j,f\}} x_{*k} w_{kj} \\ r_f &= x_{*f} - \sum_{k \in \mathbb{I} \setminus \{f,j\}} x_{*k} w_{kf} \end{aligned} \tag{S4}$$

where r_j and r_f are column vectors of size p, representing the residual activities after removing the confounding effects from the observed activities x_{*j} and x_{*f} respectively. For a bivariate distribution of two correlated variables, the affected variable is closer to the normal distribution than the contributing factor, and the higher moment statistics, skewness and kurtosis, can be used to measure the close-normality (Dodge and Yadegari 2010). As the result of the confounding effect removal, the residual activities r_j and r_f are assumed to follow a bivariate distribution, the influence direction between variables j and f can be identified by comparing the distribution shapes of r_j and r_f. In general, the removal of all confounding effects on a purely observational data is a hard issue due to the possible effects from unobserved latent factors in the domain of genomics. Under this assumption, we use a soft settings for deciding direction between j and f as following

$$Edge(j,f) = \begin{cases} j \rightarrow f \text{ and } w_{fj} := 0, & \text{if } |w_{jf}| > |w_{fj}| \text{ and } |\gamma_j| > |\gamma_f| \text{ and } |\delta_j| > |\delta_f| \\ j \leftarrow f \text{ and } w_{jf} := 0, & \text{if } |w_{jf}| < |w_{fj}| \text{ and } |\gamma_j| < |\gamma_f| \text{ and } |\delta_j| < |\delta_f| \\ j \leftrightarrow f, & \text{otherwise} \end{cases} \tag{S5}$$

where $\gamma_j = E[(r_j - \mu_{r_j})^3]/\sigma^3_{r_j}$ and $\gamma_f = E[(r_f - \mu_{r_f})^3]/\sigma^3_{r_f}$ describe the skewnesses of r_j and r_f while $\delta_j = E[(r_j - \mu_{r_j})^4]/\sigma^4_{r_j} - 3$ and $\delta_f = E[(r_f - \mu_{r_f})^4]/\sigma^4_{r_f} - 3$ describe the kurtosises respectively, where $\mu_.$ and $\sigma_.$ are the mean and standard deviation of the respective variables. That is, as describes in S5, if both moments support the same direction consistent with the stronger weight of the influence then the edge corresponding to the opposite direction is removed. Otherwise, both edges remain in the network. When we first decided dirctionality for every bidirectional edge in the GSN using the strategy S5, the precisions of directionalty inference are 0.597 ($p-value = 7.05e-06$) in BRCA and 0.559 ($p-value = 4.2e-03$) in BRCA. Then, we explored different thresholds for edge weight cut-off τ to obtain the best set of directed edges. In particular, we accept the direction with a stronger weight if one direction provides a stronger weight than the threshold τ while the opposite direction provides a weaker weight compared to τ. The edge with the weaker weight is consequently

removed from the network. The higher moment-based strategy in Equation (S5) is used to decide the direction if $|w_{fj}| \geq \tau$ and $|w_{jf}| \geq \tau$. The optimal threshold value was chosen ($\tau = 0.0391$ for BRCA and $\tau = 0.0521$ for LUAD) to maximize the fraction of correct directions (precision) in the set of recovered edges. The performance evaluation is provided in Fig. 5 in the manuscript.

Complementary to the partial correlation selection, the partial higher moment strategy provides an influence direction between two correlated variables based on their distribution shapes if the dependency direction is not resolved by the SPCS. Particularly, this strategy is proposed to remove false directed-edges from the initial partial correlation network. To evaluate the robustness of our directionality inference, we tested the reproductivity of the inferred directions by performing a bootstrapping analysis. This analysis was done by repeatedly taking 80% of samples at random without replacement and assigning a direction for each edge using the higher moment-based strategy. Of 3,987 (BRCA) and 2,685 (LUAD) edge directions that were resolved in the presenting results, the reproductivity of each edge direction is tested 100 times during the random sampling. We obtained 83.06% consistent, 2.7% inconsistent and 14.24% unsolved directions for total 3,968×100 decisions in the bootstrapping analysis on BRCA, and 84.04% consistent, 2.8% inconsistent and 13.17% unsolved directions for 2,685×100 decisions in the same analysis on LUAD. These results are summarized in Fig. S1.

S1.4 Normalization of incoming and outgoing effects

The magnitudes of the effects in the network may provide valuable information to prioritize the candidate associations of genes and MutStates with underlining biological processes because the edge weights denote the contribution scores from influence factors to their affected targets. Hence, it is reasonable to bring the total incoming and outgoing effects of nodes to the same range in the GSN. The total incoming effect on each node was attempted to be normalized into the l_1 norm constraint in Equation (S2). However, the total outgoing effects are free from normalization. Moreover, an additional update described in Equation (S5) was performed to remove edges from the initial network obtained by the SPCS model in Equation (S2).

We adopted a matrix normalization technique, alternate scaling (Sinkhorn and Knopp 1967), to rescale columns and rows of the weight matrix W into the unit l_1 norm ball. This procedure begins with rows in which each is mapped into the unit ball. Then do the same operation on columns, then on rows, and so on, until the sequence of matrices converges. The absolute difference of two consequence updates, by rows (W_{rows}) and by columns ($W_{columns}$), is used as the convergence criterion such that $\|W_{rows} - W_{columns}\|_F < 10^{-15}$. In the cases of the BRCA and LUAD analysis, the convergence was achieved after only 5 and 6 iterations, respectively. Note that this normalization increases the sparsity of the GSN since every column and row of W is iteratively mapped onto the l_1 norm space ($\|w_{k*}\|_{l_1} \leq 1$ for k-th row and $\|w_{*k}\|_{l_1} \leq 1$ for k-th column of W) which rescales the weights to lower values, even assigning zero weights to weak associations during the iterative procedure.

S1.5 Schema for generating observational data

To compare the performance of GeneSigNet with Regression tree (Huynh-Thu et al. 2010), GeneNet (Opgen-Rhein and Strimmer 2007) and LiNGAM (S. Shimizu et al. 2006) methods, we further implemented the data simulation schema provided in LiNGAM (S. Shimizu et al. 2006) to generate a data set for dependent variables. The assumption is that the observed data was generated from a process with the properties that the variables v_k, $k \in \{1, \ldots, p\}$ are arranged in an influence order, such that no later variable influences in any early variables and the value assigned to each variable

v_k is a linear function of the values assigned already to the earlier variables, plus a noise term e_k (external influence) drawn from non-Gaussian distributions as following equation

$$
\begin{aligned}
v_1 &= e_1 \\
v_2 &= w_{21} \cdot v_1 + e_2 \\
v_3 &= w_{31} \cdot v_1 + w_{32} \cdot v_2 + e_3 \\
\cdots &\qquad \cdots \qquad \cdots \\
v_p &= w_{p1} \cdot v_1 + w_{p2} \cdot v_2 + \cdots + w_{pp-1} \cdot v_{p-1} + e_p
\end{aligned}
\tag{S4}
$$

where w_{ij} denotes the weight of effect from a variable v_j on its target variable v_i. The recursive process (S4) can be represented graphically by a directed acyclic graph (DAG) where nodes are ordered in such way that no node has an edge that points to a node earlier in the order. This also implies that the adjacency matrix has only zeros on and above the diagonal. For generating a sparse DAG, non-zero weights w_{ij} were randomly scattered on the strictly lower triangular part of a zero matrix and the row index of the matrix denotes the influence other of the variables in the data generation process. That is, the samples of the seed variables in the order were first generated from standard normal distribution, then subsequently passing their influences through their down stream variables in the directed path to generate samples for the other variables.

References

Opgen-Rhein, R. and K. Strimmer (Aug. 2007). "From correlation to causation networks: a simple approximate learning algorithm and its application to high-dimensional plant gene expression data". In: *BMC Syst Biol* 1, p. 37.

Shimizu, S. et al. (2006). "A linear non-Gaussian acyclic model for causal discovery." In: *Journal of Machine Learning Research* 7.10.

Friedman, N. et al. (2000). "Using Bayesian networks to analyze expression data". In: *J Comput Biol* 7.3-4, pp. 601–620.

Tsagris, M. (Oct. 2019). "Bayesian network learning with the PC algorithm: an improved and correct variation". In: *Applied Artificial Intelligence* 33.2, pp. 101–123.

Scutari, M., C. Vitolo, and A. Tucker (Feb. 2019). "Learning Bayesian networks from big data with greedy search: computational complexity and efficient implementation". In: *Statistics and Computing* 9.5, pp. 1095–1108.

Yuan, Y., C. T. Li, and O. Windram (Apr. 2011). "Directed partial correlation: inferring large-scale gene regulatory network through induced topology disruptions". In: *PLoS One* 6.4, e16835.

Huynh-Thu, V. A. et al. (Sept. 2010). "Inferring regulatory networks from expression data using tree-based methods". In: *PLoS One* 5.9.

Jensen, F. V. and T. D. Nielsen (2007). *Bayesian networks and decision graphs.* Vol. 2. Springer.

Peng, J. et al. (June 2009). "Partial Correlation Estimation by Joint Sparse Regression Models". In: *J Am Stat Assoc* 104.486, pp. 735–746.

Yu, D. et al. (Mar. 2017). "Enhanced construction of gene regulatory networks using hub gene information". In: *BMC Bioinformatics* 18.1, p. 186.

Fujikoshi, Y., V. V. Ulyanov, and R. Shimizu (2011). "Multivariate statistics: High-dimensional and large-sample approximations". In: *Book* 760.

Tibshirani, R. (May 1996). "Regression shrinkage and selection via the lasso". In: *Journal of the Royal Statistical Society: Series B (Methodological)* 58.1, pp. 267–288.

Akaike, H. (1974). "A new look at the statistical model identification". In: *IEEE transactions on automatic control* 19.6, p. 716.

Pearl, J. (2019). “The seven tools of causal inference, with reflections on machine learning”. In: *Communications of the ACM* 62, pp. 54–60.

Chu, T. et al. (June 2003). “A statistical problem for inference to regulatory structure from associations of gene expression measurements with microarrays”. In: *Bioinformatics* 19.9, pp. 1147–1152.

Glymour, C., K. Zhang, and P. Spirtes (2019). “Review of Causal Discovery Methods Based on Graphical Models”. In: *Front Genet* 10, p. 524.

Dodge, Y. and I. Yadegari (2010). “On direction of dependence”. In: *Metrika* 72.1, p. 139.

Wiedermann, W. and J. Sebastian (2020). “Direction Dependence Analysis in the Presence of Confounders: Applications to Linear Mediation Models Using Observational Data”. In: *Multivariate Behav Res* 55.4, pp. 495–515.

Sinkhorn, R. and P. Knopp (1967). “Concerning nonnegative matrices and doubly stochastic matrices”. In: *Pacific Journal of Mathematics* 21.2, pp. 343–348.

Lachmann, A. et al. (Oct. 2010). “ChEA: transcription factor regulation inferred from integrating genome-wide ChIP-X experiments”. In: *Bioinformatics* 26.19, pp. 2438–2444.

S2 Supplementary Table

	Gene - Signature edges	Gene - Gene edges	AD of Signatures	AD of Genes
BRCA	964	66015	0.0311	0.0135
LUAD	325	78225	0.0111	0.0132

Table S1: **Topological information of the inferred networks.** Gene-Signature edges: Number of edges inferred between signatures and genes, and among signatures; Gene - Gene edges: Number of inferred edges among genes; AD of Signatures: Average degrees of the signatures; AD of Genes: Average of degrees of the genes.

S3 Supplementary Figures

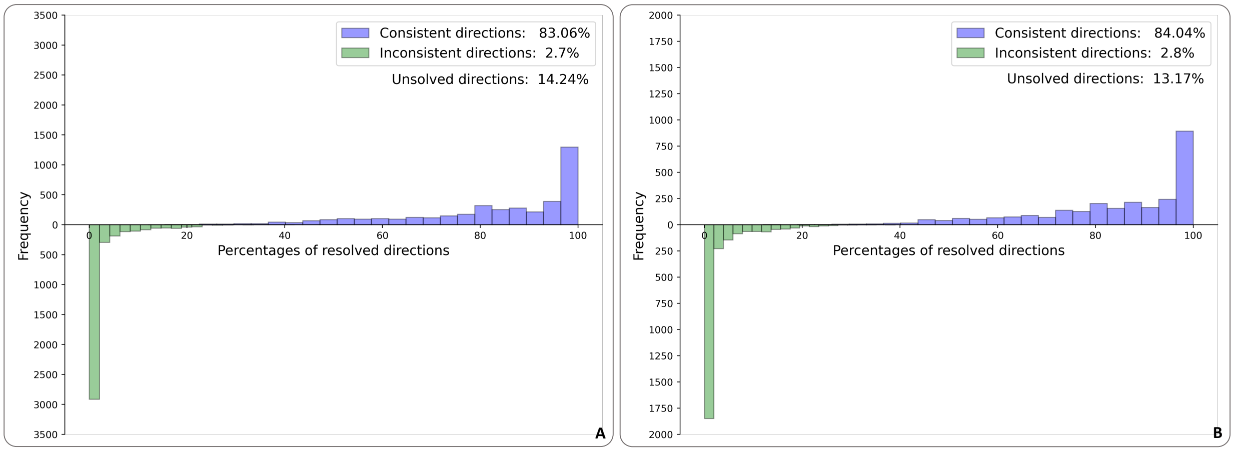

Figure S1: **Reproductivity of directionality inference.** The reproductivity of the inferred directions assigned by the partial higher moment-based strategy. The numbers of consistent and inconsistent assignments during 100 times resampling are shown in the histograms **(A)** BRCA and **(B)** LUAD. The horizontal axis represents the percentages of resolved directions during the resampling while the vertical axis represents the numbers of consistently and inconsistently assigned directions. For example, out of 3,987 edge directions were tested in the bootstrap analysis for BRCA, the consistent percentages of 1,295 directed edges fall in the interval [97%,100%] (the last bin of blue histogram) while the inconsistent percentages of 2,916 directed edges fall in the interval [0%,2%] (the first bin of green histogram). 'Consistent directions' represents the percentage of the total consistent decisions for overall the directed edges during the 100 times resampling while 'Inconsistent directions' presents the percentage of the total inconsistent decisions. 'Unsolved directions' denotes the percentage of the total unsolved decisions that the two higher moment statistics provide contradictory decisions.

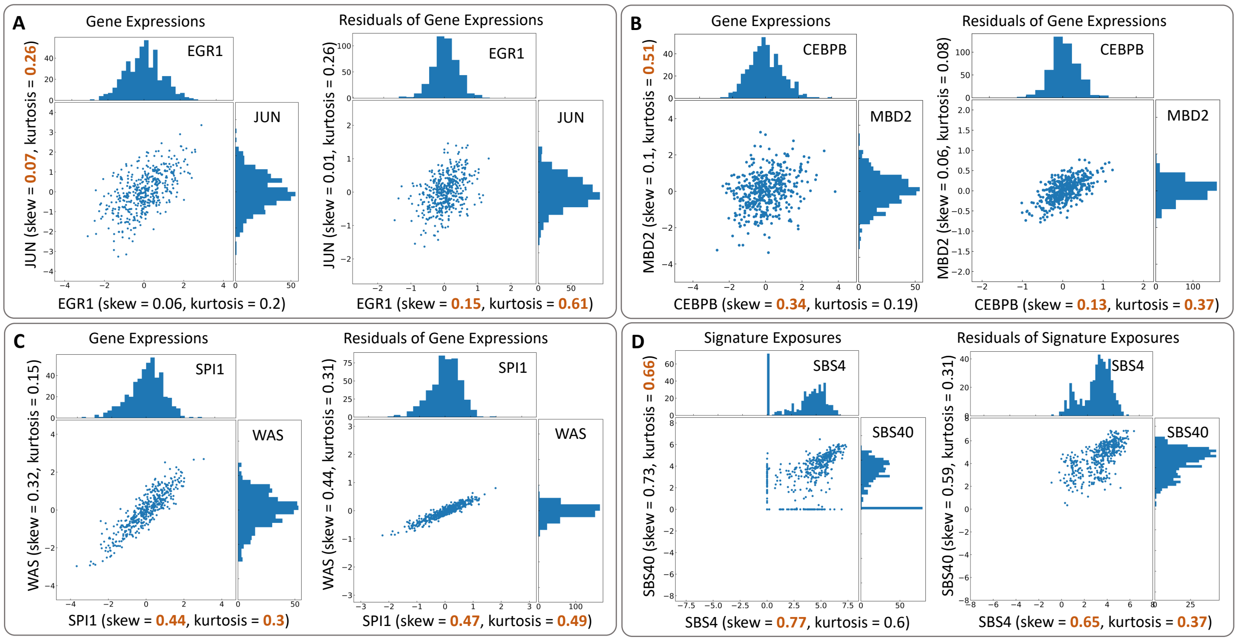

Figure S2: **Real examples for showing the necessarily of Confounding Effect Removal.** The removal of confounding effects allows the higher moment statistics to distinguish influence factor from its own target. For two random variables in each panel, the joint distribution of their observational activities is shown on the left while that of corresponding residual activities is on the right. The experimentally confirmed regulatory relations (Lachmann et al. 2010) indicate the correct directions. The values in brown highlight the influence factors. **(A)** Without removing confounding effects from expressions of EGR1 and JUN, both moments provided an incorrect direction. After removing the confounding effects, the two moments indicate the correct direction. **(B)** Before removing the effects, the skewness provide the direction from CEBPB to MBD2 while the kurtosis supports the opposite direction. However, the proposed higher moment-based strategy indicates the correct direction **(C)** Either without or with the effect removal, the two moments provide the experimentally confirmed influence in WAS from SPI1. Although the higher moment-based strategy did not suggest any changes on the directionality decision, the effect removal uncovers a stronger association between the two genes. **(D)** The two moments provide contradictory decisions without removing the confounding effects from the exposures of the two mutational signatures. However, with the removal, the inferred direction is from SBS4 to SBS40.

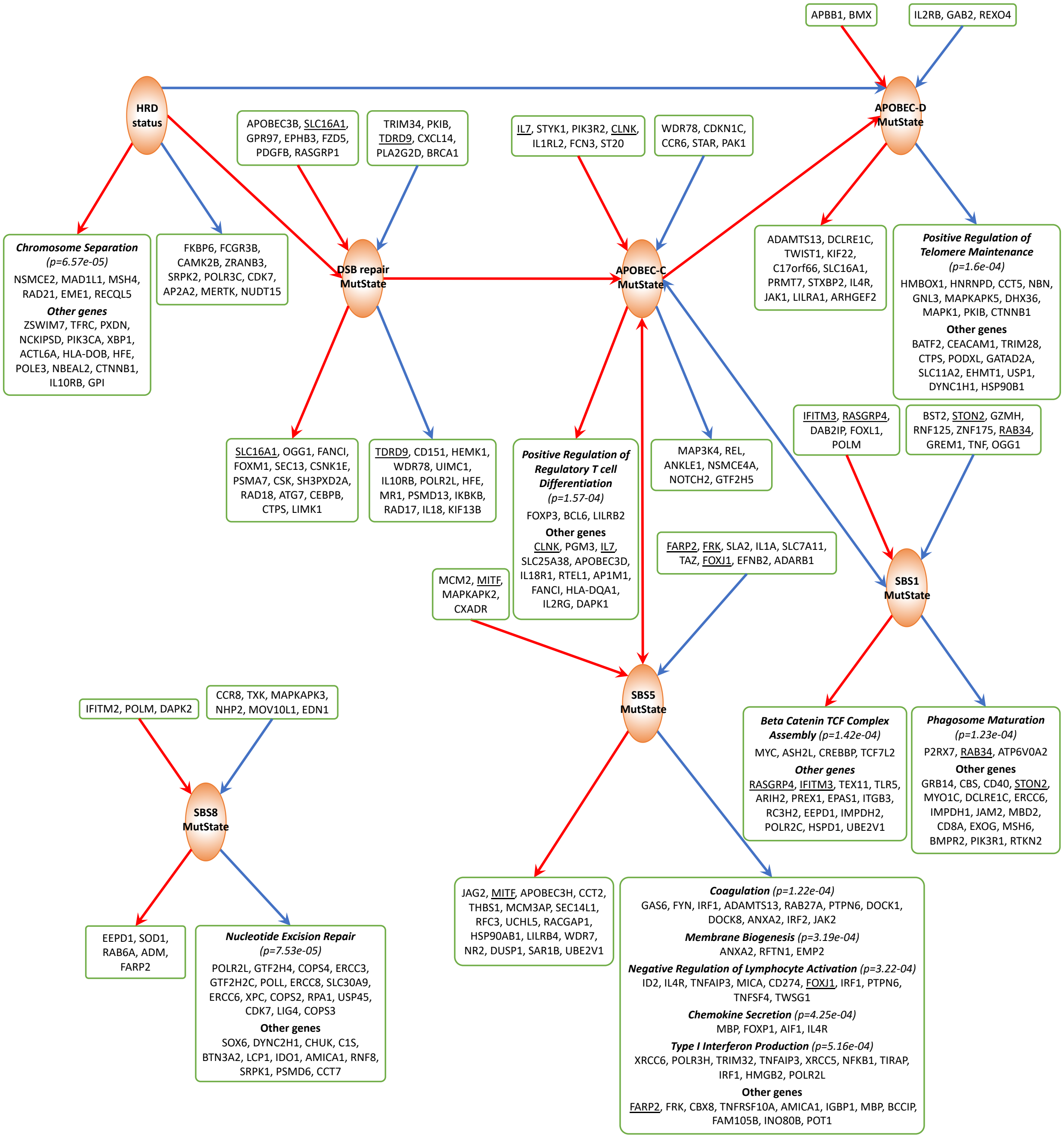

Figure S3: **Subnetwork of GSN for BRCA centered on MutStates.** Edge and node colors are as in Fig. 1 in the manuscript. If the genes adjacent to a given MutState are enriched with specific GO pathways ($q - value < 0.01$), the names of the enriched genes are provided under the pathway names in a box and the other adjacent genes with edge weight cut-off ($|w_{ij}| \geq 0.01$) are titled as 'Other genes' in the same box. If the adjacent genes are not enriched with any specific GO pathways ($q - value < 0.01$), the names of the genes with edge weight cut-off ($|w_{ij}| \geq 0.01$) are provided in a box. The complete lists of upstream and downstream sets for MutStates inferred in the analysis on the BRCA data are provided in Supplementary Table S2 in Additional file 2, and their enriched GO terms are provided Supplementary Table S4 in Additional file 3.

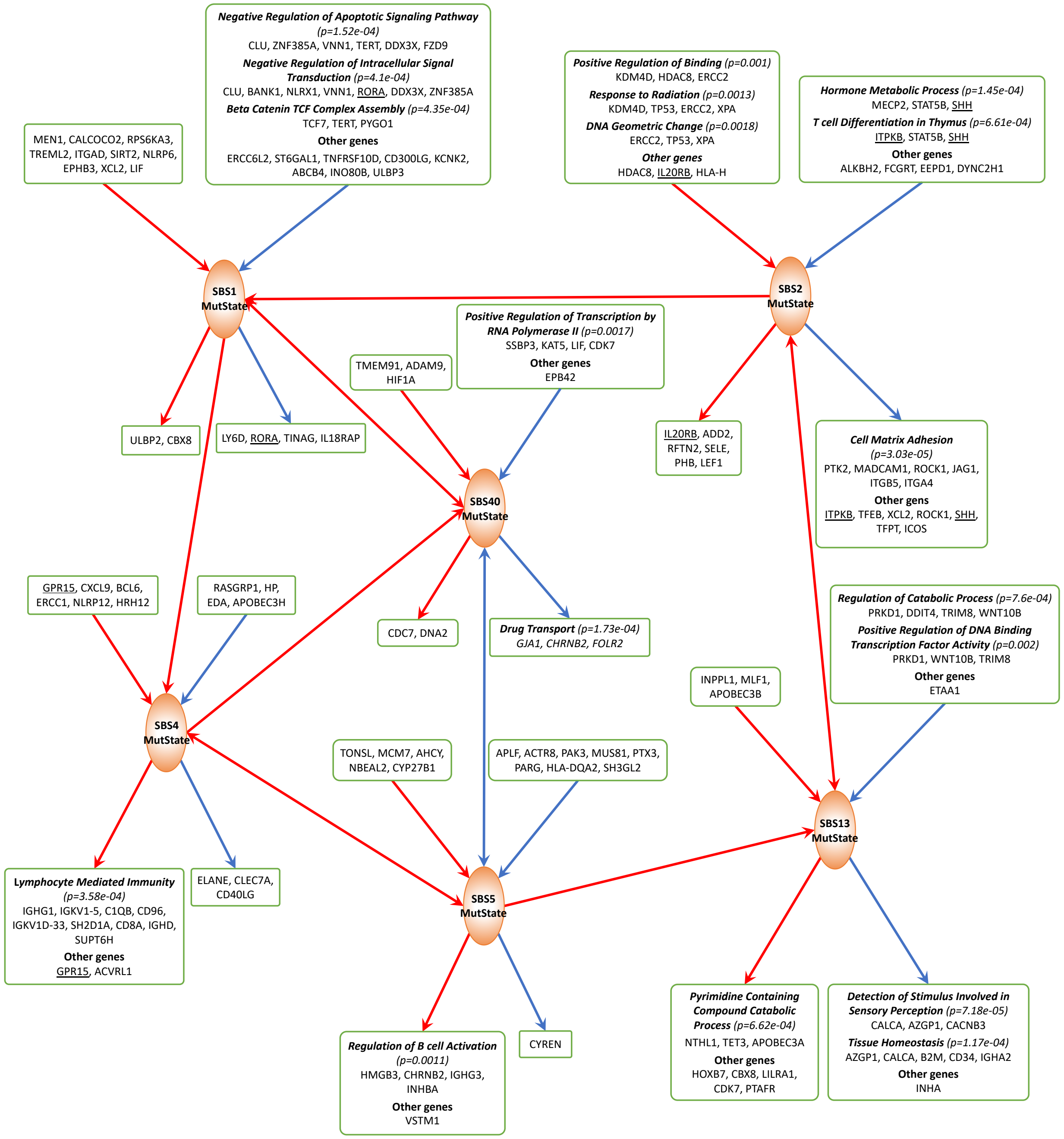

Figure S4: **Subnetwork of GSN for LUAD centered on MutStates.** The meaning of edge and node colors, and boxes is the same as in Fig. S3. The complete lists of upstream and downstream sets for MutStates inferred in the analysis on the LUAD data are provided in Supplementary Table S3 in Additional file 2, and their enriched GO terms are provided Supplementary Table S5 in Additional file 3.
